# Polarized Microscopic Analysis of Picrosirius Red Stained Salivary Gland Pathologies: An Observational Study

**DOI:** 10.1055/s-0042-1743145

**Published:** 2022-05-17

**Authors:** Rachai Juengsomjit, Ounruean Meesakul, Tawepong Arayapisit, Noppadol Larbcharoensub, Kajohnkiart Janebodin

**Affiliations:** 1 Department of Oral and Maxillofacial Pathology, Faculty of Dentistry, Mahidol University, Bangkok, Thailand; 2 Department of Anatomy, Faculty of Dentistry, Mahidol University, Bangkok, Thailand; 3 Department of Pathology, Faculty of Medicine, Ramathibodi Hospital, Mahidol University, Bangkok, Thailand

**Keywords:** salivary gland diseases, picrosirius red, polarized light, Masson's trichrome, collagen

## Abstract

**Objective** Salivary gland diseases and their pathologies may affect the glandular structure including collagen, a major stromal component, in response to tissue damage or diseases. This study aimed to examine the changes in collagens in different salivary gland diseases using polarized picrosirius red staining.

**Materials and Methods** The submandibular gland samples diagnosed as sialadenitis, chronic sclerosing sialadenitis, pleomorphic adenoma, adenoid cystic carcinoma, and mucoepidermoid carcinoma were stained with picrosirius red, Masson's trichrome, and anticollagen I staining. The quantity of collagens was examined and reported as a percentage of positive picrosirius red area. The maturity of collagens was studied with polarized light microscope and reported as a percentage of orange-red and yellow-green polarized collagens, representing the mature and immature collagens, respectively.

**Statistical Analysis** The % positive areas for picrosirius red representing the collagen amount among salivary gland diseases were analyzed by one-way analysis of variance with Tukey's test. The % orange-red and % yellow-green polarized areas representing the collagen maturity were analyzed by Kruskal–Wallis test and Mann–Whitney U test.

**Results** The malignant tumors, adenoid cystic carcinoma (29.92) and mucoepidermoid carcinoma (26.59), had higher significant percentage of positive picrosirius red area, compared with the benign tumor (14.56), chronic sclerosing sialadenitis (10.61), and sialadenitis (7.22) ( *p* < 0.05). The percentages of orange-red polarized areas are 48.07, 39.6, 62.67, 83.75, and 76.05 in sialadenitis, chronic sclerosing sialadenitis, pleomorphic adenoma, adenoid cystic carcinoma, and mucoepidermoid carcinoma, respectively. This percentage tended to increase in the benign and malignant lesions with statistical difference, compared with the inflammatory lesions ( *p* < 0.05). There was no statistical difference in the percentages of yellow-green polarized areas among various salivary gland diseases. In addition, the results of Masson's trichrome and anticollagen I staining are corresponding to that of picrosirius red among various salivary gland diseases.

**Conclusions** Polarized picrosirius red demonstrated the most amounts of collagen in the malignant lesion, and represented the different maturity of collagens in each lesion group. Studying the amounts and maturity of collagen with picrosirius red for extracellular matrix alteration in salivary gland diseases along with routine hematoxylin and eosin, Masson's trichrome, and immunohistochemistry may provide a better understanding in different salivary gland pathologies.

## Introduction

Salivary glands, which are important exocrine glands in our body, function to produce and secrete saliva. Based on their sizes and locations, major salivary glands generate 90% of total saliva and consist of parotid, submandibular, and sublingual salivary glands. Minor salivary glands produce the rest of total saliva found in lips, cheeks, palate, and tongue. 1 Patients with salivary gland diseases can suffer from pain and swelling, facial numbness and weakness, and limited mouth opening. In addition, treatments of salivary gland tumors with radiation and surgery can cause a decrease in saliva secretion, 2 leading to patients' low quality of life due to various oral complications with altered taste perception, and difficulty in swallowing and speech. 3

Regarding the histopathology, salivary gland diseases are classified into inflammations, and benign and malignant tumors. 4 Sialadenitis and chronic sclerosing sialadenitis are two salivary gland inflammations that are commonly found in submandibular salivary glands. 5 A previous study has reported the prevalence of salivary gland tumors in Thais and shown that the tumors are found the most in parotid gland, submandibular gland, and minor salivary glands. The most benign tumor found is pleomorphic adenoma. The most malignant salivary gland tumor is mucoepidermoid carcinoma, followed by adenoid cystic carcinoma of total salivary gland tumors. 6

Collagen fibers are one of the main components in stromal tissue including salivary glands. They play a role in maintaining tissue structure and influencing specific functions in different organs. 7 Once the tissue damage is occurred, the collagen fibers would be altered in their arrangement, diameter, and cross-sectional structures. Each disease may differently affect the alteration of biological levels of tissues, contributing to different characteristics of collagens. 8 9 Hence, changes in extracellular matrices including collagens may give us a hint involving the pathogenesis of salivary gland diseases.

Hematoxylin and eosin (H&E) staining is a common histological approach to study collagen fibers. Although this basic staining enables to demonstrate anatomical and morphological changes, as well as arrangement of collagens in the tissues, it limits the study of collagen reorganization and deposition. 10 In some tissues with the intense background, Masson's trichrome stain is used due to its advantage with three colors to distinguish connective tissue stoma, capsule, or extracellular matrix from the cellular component. 11 Picrosirius red staining is another simpler but useful method to study collagen arrangement. It can be quite spectacular and colorful providing results that are much more informative than routine H&E.

Sirius red stains collagens by reacting via its sulfonic acid groups, with basic groups presented in the collagen molecules. When this staining is combined with polarized microscope, it permits an easy and precise localization and characterization of the tissue components containing orientated collagen molecules. 12 Since the collagen fibrils have different arrangement directions, it leads to different refractive indices. This will cause birefringence, creating different colors. 12 13 Picrosirius red combined with the polarized light shows that the collagen bundle in a variety of tissue is arranged in a different orientation and able to reflect light. 13 14 The reflected orange-red color represents more packed (mature) collagen fibers while yellow-green color represents less packed (immature) collagen fibers. 15 16

This study aimed to investigate the histopathological changes of collagens in three different groups of salivary gland diseases—inflammation, benign tumors, and malignant tumors—using picrosirius red combined with the polarized light microscope, in parallel with H&E, Masson's trichrome, and anticollagen I immunohistochemistry. We hypothesized that each salivary gland disease demonstrated differences in the amounts and maturity of collagens, which were analyzed by birefringence from the polarized microscope. The results may provide a different observation in patterns of collagen cumulation among various salivary gland diseases for diagnosis.

## Materials and Methods

### Tissue Preparation and Staining

Submandibular salivary gland tissue samples from patients aged 25 to 80 years old, which were histologically diagnosed as sialadenitis ( *n* = 16) and chronic sclerosing sialadenitis ( *n* = 4) for salivary gland inflammation, pleomorphic adenoma for benign tumor ( *n* = 5), and adenoid cystic adenoma ( *n* = 7) as well as mucoepidermoid carcinoma ( *n* = 4) for malignant tumors, were collected. Tissue sections from paraffin blocks were cut for 5 µm thickness. H&E, picrosirius red, and Masson's trichrome staining were performed to study the tissue morphology and collagen fibers, respectively.

Picrosirius red staining was conducted following the manufacturer's instruction kit (Bio-optica, Milano, Italy). Briefly, the section was covered by the picrosirius red solution (0.1% of sirius red in saturated aqueous picric acid) and incubated for 30 minutes at room temperature. Then, the stained sections were washed once and stored in distilled water, with rocking motion for overnight, to remove nonspecific binding. The next day, the sections were dehydrated with series of different concentrations of alcohols (60, 70, 80, 90, and 100%) and xylene twice for 1 minute each. Then, stained sections were mounted with Permount Mounting Media prior to analysis.

Masson's trichrome staining was conducted following the manufacturer's instruction kit (Abcam, ab150686). Briefly, the sections were incubated in preheated Bouin's solution for 60 minutes, cooled for 10 minutes, and then rinsed in water. Then, slides were incubated in Weigert's iron hematoxylin for 5 minutes and then rinsed in water. Next, slides were incubated in Biebrich Scarlet/Acid Fuchsin solution for 15 minutes and then rinsed in water. Sections were differentiated in phosphomolybdic/phosphotungstic acid solution for 15 minutes before being incubated in Aniline Blue for 10 minutes and rinsed in water. Then, slides were incubated in acetic acid for 5 minutes. Finally, stained slides were dehydrated and mounted.

### Immunohistochemistry

Tissue sections were deparaffinized and rehydrated using xylene and ethanol (100% and 95%), respectively. Then, endogenous peroxidase was blocked by 3% hydrogen peroxide for 10 minutes before the sections were washed with distilled water twice for 5 minutes each. For antigen retrieval, citrate buffer (pH 6.0, 98) heated under scientific microwave was used for 20 minutes. Sections were washed with phosphate buffer saline (PBS) pH 7.6 mixed with 0.1% tween for three times, 5 minutes each. Next, nonspecific endogenous binding was blocked by 200 µL of 5% bovine serum albumin for 30 minutes at room temperature. Primary rabbit antihuman collagen type I monoclonal IgG antibody (Abcam, ab138492) (0.5 µg/mL) at 1:1,500 was used and incubated for 2 hours at room temperature. Then, stained slides were washed with PBS (pH 7.6) mixed with 0.1% tween for three times, 5 minutes each. Secondary goat antirabbit IgM antibody conjugated with peroxidase (Dako, EnVision+ Dual Link System-HRP) was applied for 30 minutes at room temperature before washing with PBS (pH 7.6) mixed with 0.1% tween for three times, 5 minutes each. 3,3'-Diaminobenzidine chromogen was used for 5 minutes to develop the signal and then the final wash was performed under the running tap water. The stained sections were counterstained with hematoxylin, dehydrated, and mounted with Permount. The specificity of primary antibody was confirmed by the positive staining result in normal salivary gland connective tissue septa as positive control. Sections stained with Rb IgG were used as the negative control.

### Imaging by Polarized Microscope

Stained tissue sections were imaged by the polarized microscope (Nikon ECLIPSE E400 POL) with the camera (Nikon digital camera D5100). The areas of granulation tissue were imaged in salivary gland inflammation whereas those of epithelial neoplastic islands and capsule were selected in benign and malignant salivary gland tumors. All images from picrosirius red combined with polarized were recorded for further analyses.

### Image Interpretation

Intrapersonal and interpersonal reliabilities were examined by the paired *t* -test to represent insignificant statistical difference before further analysis. Amounts of collagen were analyzed by using ImageJ software to compare the percentage of positive picrosirius red area per total area (% area of picrosirius red). At least five areas were chosen from each stained section under 100× magnification.

The stained collagens were observed under a polarized microscope. The polarized images represented different colors and thickness of collagens, which indicate the difference in maturity of collagens. Digital RGB—red, green, blue—images of collagens were captured in five areas per section and subjected to ImageJ software. The areas of red-orange and yellow-green colors were quantitated to represent mature and immature collagens, respectively. Each color was binarized by using a fixed threshold value and the area of positive pixels was determined. Total red-orange and yellow-green areas from each section were demonstrated as percentage of area (% of polarized area) relatively to the control, which is the normal submandibular salivary gland tissues.

### Statistical Analysis

All data were analyzed using program SPSS— *Statistical Package for the Social Sciences* —version 18.0 and demonstrated as mean values from at least 20 areas (mean ± standard deviation) in each group of salivary gland diseases. One-way analysis of variance was used to demonstrate the differences among groups, and post-hoc multiple comparison with Tukey's test was used to determine the differences between groups at the 95% confidential interval. The % orange-red and % yellow-green polarized areas representing the collagen maturity among salivary gland diseases were analyzed by Kruskal–Wallis test and Mann–Whitney U test to determine the differences among groups at the 95% confidential interval.

## Results

### H&E Staining

**Fig. 1:**
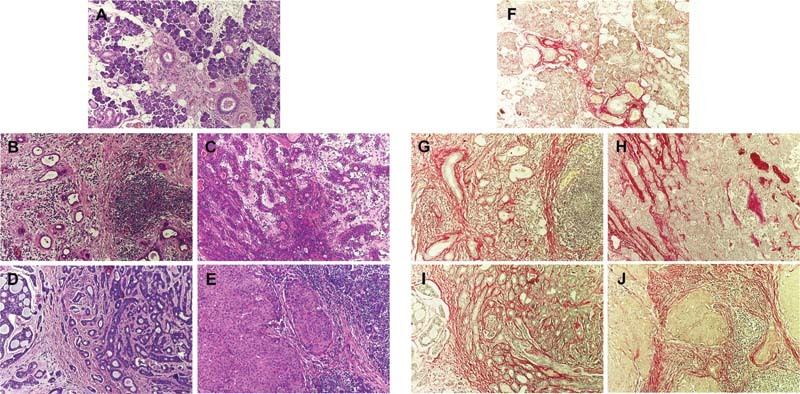
Representative hematoxylin and eosin and picrosirius red staining of different salivary gland diseases. ( **A, F** ) Sialadenitis with lymphocyte infiltration in glandular parenchyma demonstrating less amounts of collagen, which was mostly found in the area of connective tissue septa with the ductal structure. ( **B, G** ) Chronic sclerosing sialadenitis with lymphocyte infiltration in atrophic gland tissue exhibiting more dispersed collagens relating to inflammatory cells and atrophy acini. ( **C, H** ) Pleomorphic adenoma showing duct-like structures and myoepithelial neoplastic cells in a myxoid stroma displaying collagen fibers in stromal tissue and close to myoepithelial neoplastic cells. ( **D, I** ) Adenoid cystic adenoma showing the tumor region with the cribriform pattern surrounded by fibrous connective tissue. ( **E, J** ) Mucoepidermoid carcinoma showing fibrous tissue surrounding the tumor mass containing the island of epidermoid cells with mucus containing cells, x100 magnification.

The histopathology of sialadenitis exhibited lymphocyte infiltration in connective tissue septa and glandular parenchyma, indicating presence of inflammation ( Fig. 1A ). Chronic sclerosing sialadenitis showed lymphocyte infiltration with atrophy of glandular tissue and more fibrous tissues compared with sialadenitis ( Fig. 1B ). Pleomorphic adenoma was characterized by duct-like structures and myoepithelial neoplastic cells in a myxoid stroma ( Fig. 1C ). Adenoid cystic carcinoma showed the tumor region with the cribriform pattern surrounded by fibrous tissue ( Fig. 1D ). Mucoepidermoid carcinoma represented the fibrous tissue surrounding the tumor mass containing the island of epidermoid (epithelial neoplastic) cells with mucus-containing cells ( Fig. 1E ).

### Picrosirius Red Staining

Sialadenitis ( Fig. 1F ) demonstrated less amounts of collagen, which was mostly found in the area of connective tissue septa with the ductal structure. Chronic sclerosing sialadenitis ( Fig. 1G ) exhibited more dispersed collagens relating to inflammatory cells and atrophy acini. Pleomorphic adenoma ( Fig. 1H ) displayed collagen fibers in stromal tissue and close to myoepithelial neoplastic cells. Malignant salivary gland tumors ( Fig. 1I and J ) exhibited more amounts and distribution of collagen fibers, compared with benign salivary gland tumors. Interestingly, clusters of tumor cells were surrounded by collagen fibers.

### Masson's Trichrome Staining

**Fig. 2:**
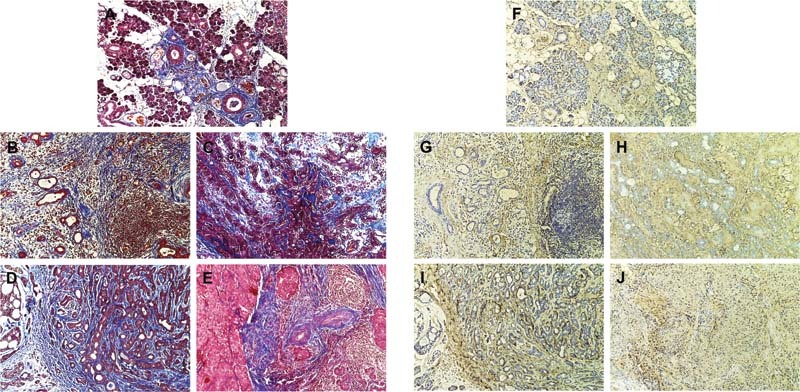
Representative Masson's trichrome and anticollagen I staining of different salivary gland diseases. The figures were imaged from the same area from Fig. 1 . ( **A, F** ) Sialadenitis exhibiting less amounts and disperse collagen fibers with positive anticollagen I. ( **B, G** ) Chronic sclerosing sialadenitis showing more positive staining of Masson's trichrome and anticollagen I observed especially in the area surrounding lymphocyte infiltration and atrophy glandular tissues. ( **C, H** ) Pleomorphic adenoma showing more packed collagens that were positive to Masson's trichrome and anticollagen I running parallel to benign neoplastic cells in connective tissue stroma. ( **D, E** ) Adenoid cystic carcinoma. ( **I, J** ) Mucoepidermoid carcinoma showing tumor islands surrounded by collagen fibers. More amounts of collagen fibers were seen, compared with benign salivary gland tumors, x100 magnification.

Corresponding to picrosirius red staining, Masson's trichrome showed the same pattern of collagen distribution in each salivary gland disease. This histochemical stain displayed three colors of red, black, and blue to indicate cellular cytoplasm, nuclei, and collagen fibers, respectively. Sialadenitis ( Fig. 2A ) and chronic sclerosing sialadenitis ( Fig. 2B ) exhibited less amounts and disperse collagen fibers, compared with salivary gland tumors. Pleomorphic adenoma showed more packed collagens running parallel to benign neoplastic cells ( Fig. 2C ). Malignant salivary gland tumor cells were surrounded by collagen fibers in both adenoid cystic carcinoma and mucoepidermoid carcinoma ( Fig. 2D and E ). More amounts of collagen fibers were seen, compared with benign salivary gland tumors.

### Immunohistochemistry

Corresponding to picrosirius red and Masson's trichrome staining, the same location of connective tissue displayed positive anticollagen I staining but various intensities in all salivary gland diseases. However, not all of the areas positive to picrosirius red and Masson's trichrome showed the positive stain of anticollagen I. Compared with sialadenitis ( Fig. 2F ), more positive staining of anticollagen I was observed in chronic sclerosing sialadenitis especially in the area surrounding lymphocyte infiltration and atrophy glandular tissues ( Fig. 2G ). Pleomorphic adenoma demonstrated the positive area of anticollagen I in connective tissue stroma ( Fig. 2H ). Noticeably, anticollagen I stain was slightly intense in the connective tissue close to the tumor cells in adenoid cystic carcinoma ( Fig. 2I ) and mucoepidermoid carcinoma ( Fig. 2J ).

### Polarized Picrosirius Red Staining

**Fig. 3:**
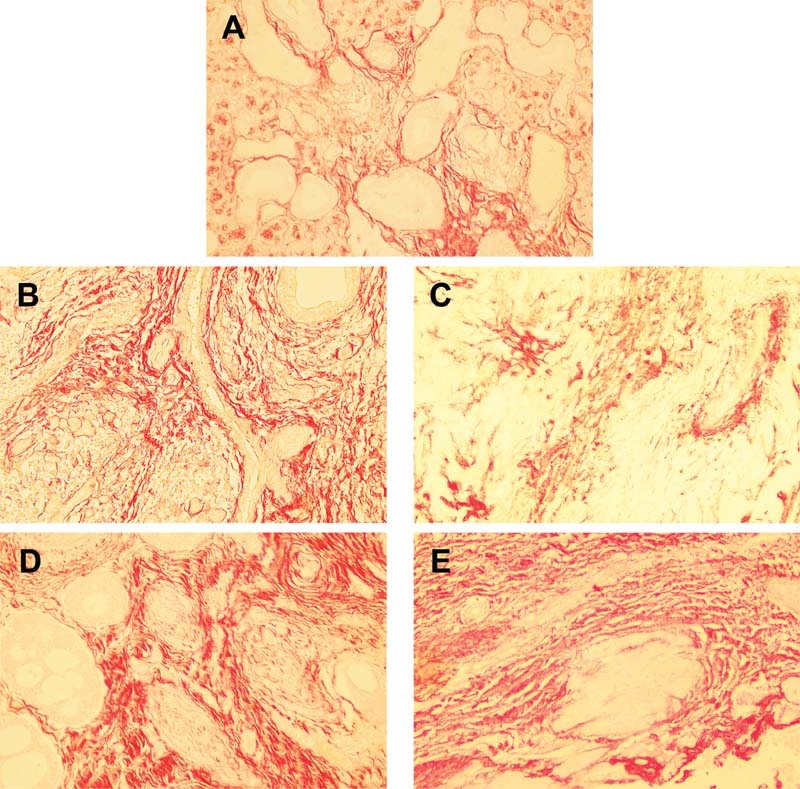
Representative picrosirius red staining of different salivary gland diseases under light microscope. Differences in amounts of positive red staining were shown in each disease. Sialadenitis ( **A** ) and chronic sclerosing sialadenitis ( **B** ) showed less amounts and disperse distribution of collagen, compared with salivary gland tumors ( **C** – **E** ). Malignant salivary gland tumors ( **D–E** ) exhibited more amounts and compacted collagen, compared with benign salivary gland tumors ( **C** ). A, sialadenitis; B, chronic sclerosing sialadenitis; C, pleomorphic adenoma; D, adenoid cystic adenoma; E, mucoepidermoid carcinoma, x100 magnification.

Picrosirius red staining showed differences in the amounts of positive red staining in each salivary gland disease, indicating various amounts and patterns of collagen. Sialadenitis ( Fig. 3A ) and chronic sclerosing sialadenitis ( Fig. 3B ) demonstrated less amounts and disperse distribution of collagen, compared with salivary gland tumors. Nevertheless, malignant salivary gland tumors ( Fig. 3D, E ) exhibited more amounts and compact arrangement of collagen, compared with benign salivary gland tumors ( Fig. 3C ).

**Fig. 4:**
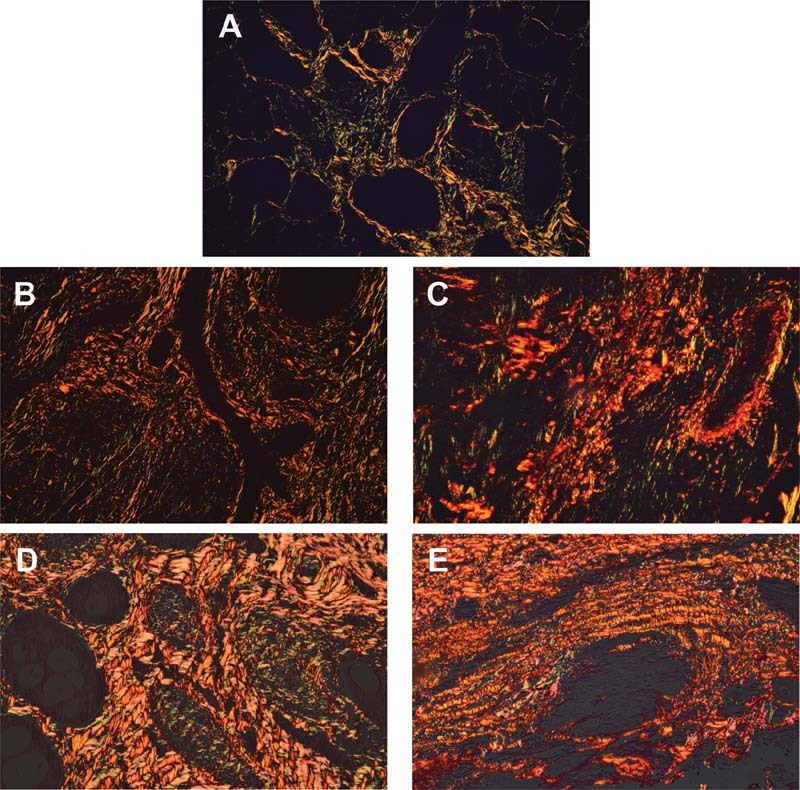
Representative picrosirius red staining of different salivary gland diseases under polarized light microscope. The figures were imaged from the same area from Fig. 1 . Each disease showed a various combination of different collagen fibers. Sialadenitis showed the distribution of more yellow-green thin fibers (less mature collagens) ( **A** ). Sclerosing condensing sialadenitis showed a mixture of yellow-green and orange-red thin fibers ( **B** ). Benign and malignant tumors including the area surrounding the tumor clusters exhibit more orange-red thick fibers (more mature collagens) ( **C–E** ). A, sialadenitis; B, chronic sclerosing sialadenitis; C, pleomorphic adenoma; D, adenoid cystic adenoma; E, mucoepidermoid carcinoma, x100 magnification.

Each salivary gland disease showed variable color ranges and thickness, which represented the combination of different collagen fibers ( Fig. 4A–E ). The center of inflammation in sialadenitis showed the distribution of more yellow-green with less orange-red thin fibers, representing more immature collagen production ( Fig. 4A ). Meanwhile, sclerosing condensing sialadenitis exhibited an increase in orange-red thin fibers ( Fig. 4B ). Conversely, the center of benign and malignant tumors including the area surrounding the tumor clusters exhibited more orange-red and thick fibers, indicating more mature collagens ( Fig. 4C–E ).

### Image Analysis

**Fig. 5:**
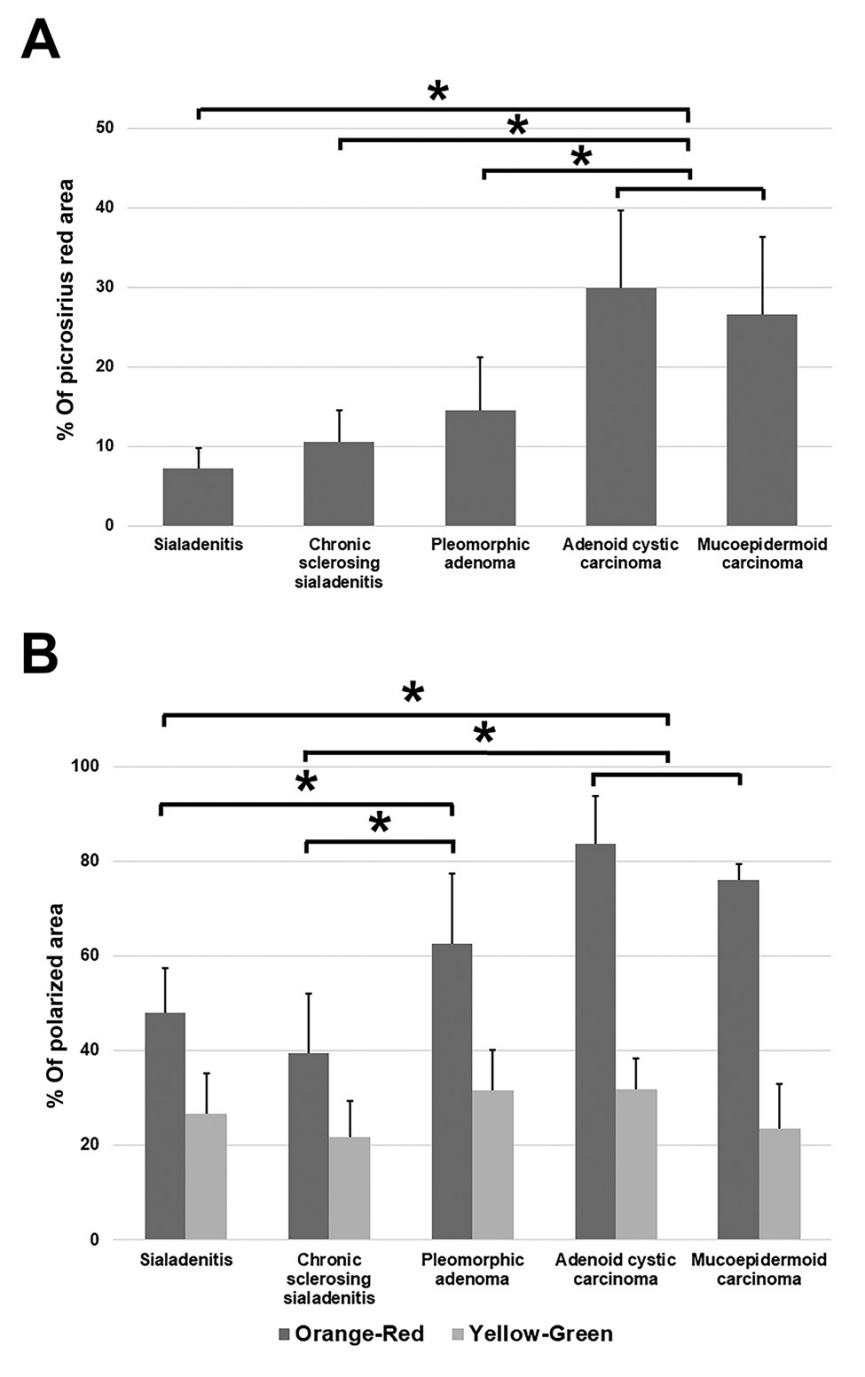
The bar graph represents different percentage areas of positive picrosirius red, and different percentages of polarized areas of orange-red and yellow-green color among different salivary gland diseases. ( **A** ) Pleomorphic adenoma, chronic sclerosing sialadenitis, and sialadenitis showed 14.56, 10.61, and 7.22% of positive areas for picrosirius red staining, respectively. Malignant salivary gland tumors including adenoid cystic carcinoma and mucoepidermoid carcinoma presented the higher percentage areas (29.92 and 26.59%) of positive picrosirius red. The data showed the significant difference of the positive picrosirius red areas in malignant tumors, compared with other salivary gland diseases ( *p* < 0.05). Percent (%) of Picrosirius Red Area is represented as mean ± standard deviation (SD); * *p* < 0.05. ( **B** ) The % orange-red polarized areas were 48.07, 39.6, 62.67, and 81.55 in sialadenitis, chronic sclerosing sialadenitis, pleomorphic adenoma, and malignant tumors, respectively. There is no statistical difference between sialadenitis and chronic sclerosing sialadenitis in % orange-red polarized areas. Malignant salivary gland tumors and pleomorphic adenoma show a significant increase in % orange-red polarized areas (mature collagens), compared with both inflammatory salivary gland diseases. The malignant tumors show an insignificant difference, compared with pleomorphic adenoma. The comparison of % yellow-green polarized areas (immature collagens) among salivary gland diseases (26.62, 21.83, 31.73, 31.86, and 23.66 in sialadenitis, chronic sclerosing sialadenitis, pleomorphic adenoma, adenoid cystic carcinoma, and mucoepidermoid carcinoma, respectively) does not show any statistical differences. Percent (%) of Polarized Area is represented as mean ± SD; * *p* < 0.05.

The semiquantitative analysis was performed to quantify the percentage positive area for picrosirius red to present the amounts of collagen ( Fig. 5A ). Benign salivary gland tumor, chronic sclerosing sialadenitis, and sialadenitis showed 14.56, 10.61, and 7.22% of positive area for picrosirius red staining, respectively. Intriguingly, malignant salivary gland tumors including adenoid cystic carcinoma and mucoepidermoid carcinoma presented the higher percentage area (29.92 and 26.59%, respectively) of positive picrosirius red. The significantly statistically higher positive picrosirius red areas in malignant tumors were shown, compared with other salivary gland diseases ( *p* < 0.05).

To determine the collagen maturity in each disease, % orange-red and % yellow-green polarized areas were analyzed and presented to imply mature and immature collagens, respectively ( Fig. 5B ). Regarding % orange-red polarized area, there was no statistical difference between sialadenitis (48.07%) and chronic sclerosing sialadenitis (39.6%). Meanwhile, adenoid cystic carcinoma (83.75%), mucoepidermoid carcinoma (76.05%), and pleomorphic adenoma (62.67%) exhibited increases in % polarized area with significant statistical difference compared with both inflammatory salivary gland diseases, suggesting a presence of more collagen maturity. However, the malignant tumors showed insignificant statistical difference when compared with pleomorphic adenoma. Moreover, there was no difference in % orange-red polarized area in two types of malignant tumors. Interestingly, the comparison of % yellow-green polarized area that indicates immature collagens among salivary gland diseases did not show any statistical differences.

## Discussion

Collagen fiber, a major composition in connective tissue stroma, functions to give strength to tissues and also protects body structures from spreading of pathogens, toxins, and cancerous cells. 17 Changing of collagen in response to tissue damage or diseases in various organs has been reported. 9 18 Therefore, the pathology of salivary gland diseases including inflammation, and benign and malignant tumors may also influence a distinctive alteration of collagen fibers, suggesting the disease's biological behavior. In this study, we investigated the collagen fibers in sialadenitis, sclerosing sialadenitis, pleomorphic adenoma, adenoid cystic carcinoma, and mucoepidermoid carcinoma due to the high prevalence of salivary gland diseases in Thai population.

H&E, Masson's trichrome, and anticollagen I staining combined with polarized picrosirius red staining were used to investigate the amount and maturity of collagen in salivary gland diseases. In this study, Masson's trichrome and picrosirius red staining displayed the similar results in amounts of collagen fibers among various salivary gland diseases. In addition, the immunohistochemistry of collagen type I was conducted to investigate the presence of collagen type I, which is more mature collagen. Intriguingly, salivary gland tumors showed more positive staining of anticollagen type I, especially the area close to tumor cells, suggesting the presence of more mature collagens. Nevertheless, certain areas of connective tissue that were positively stained for Masson's trichrome and picrosirius red showed a negative result of anticollagen I. Those locations might contain a different type of collagen such as collagen type III, which represents immature collagen fibers.

This study aimed to use the advantage of polarized picrosirius red to examine the characteristics of collagen in inflamed salivary gland and tumors. Although the picrosirius red with polarized light microscope is not regularly used in the clinical work, some evidences about the superiority of picrosirius red have been reported. For instance, compared with Masson's trichrome, picrosirius red with polarization is very accurate to determine the very thin collagen fibers. This would be beneficial to decrease an opportunity to underestimate the collagen content in the sample. Moreover, Masson's trichrome exhibits a tendency to fade and has more steps in its staining protocol. 19

Regarding the picrosirius red stained tissues of different salivary gland diseases, malignant salivary gland tumors exhibited significantly higher value of % positive red area compared with other salivary gland diseases ( *p* < 0.05), implying more collagen production. This phenomenon may be explained by the body mechanism attempting to limit the invasion of inflammation or cancer cells. 20 21 Moreover, fibrous stroma may be generated from the process of epithelial–mesenchymal interaction to exchange nutrients and waste products in the tumor progression. 22

Picrosirius red staining enables to stain collagen fibers and indicates the presence of collagen amounts in each lesion; however, this staining is still limited for indicating the maturity of collagen fibers. Consequently, the picrosirius red staining with polarized microscope is applied to distinguish the different color ranges between orange-red and yellow-green to indicate the mature and immature collagen fibers, respectively. This is considered as another advantage of picrosirius red to indirectly examine the collagen maturity, which would be further confirmed by special antibody staining. Several studies using polarizing picrosirius red suggested differences in collagen affecting their biological characteristics of oral lesions. 13 14 16 23

Our study has demonstrated that salivary gland inflammation and tumors generated more collagen fibers. This might imply the production of fibrosis for tissue healing or protective mechanism. 24 Regarding polarized picrosirius red stain, a majority of collagen in sialadenitis showed a mixture of more yellow-green and less orange-red thin fibers. Moreover, there was a gradual change from immature to mature collagens as inflammation progressed in sclerosing chronic sialadenitis, indicating that the chronic inflammation influences collagen fibers in the connective tissue stroma. This observation may be supported by the study of Hirshberg et al, where thick and mature collagens were mainly found in the wall of odontogenic keratocysts with a presence of dense inflammation. 25

The percent of orange-red area tends to be higher in malignant salivary gland tumors. This value significantly differs from that of inflamed glands, but it does not when compared with benign salivary gland tumors. Corresponding to the nature of this tumor type that has a slow progression when compared with other types of oral cancer, 26 this may be explained by the attempt in producing mature collagens to inhibit the invasion of cancerous cells. 27 However, this is in contrast to the study done by Allon et al, where predominant yellow-green collagen fibers in adenoid cystic carcinoma were observed. Mucous extravasation phenomenon as control and benign salivary gland tumors exhibited significantly more mature orange-red collagens, compared with malignant salivary gland tumors. 23 This might be explained by the longstanding nature of the lesion, different location of salivary glands, and also different patients' ages in our study.

In addition to the epithelial–mesenchymal interaction, the morphological alteration of pathological salivary glands has been recently reported involving in the epithelial–mesenchymal transition (EMT). 28 EMT is occurred when epithelial cells transform themselves by loss of intercellular junctions and cellular polarity to have cellular properties mimicking to connective tissue cells. 29 This process plays an important role in connective tissue stromal changes due to inflammation and tumor metastasis. 8 20 30 For instance, collagens in sialadenitis might be formed by the salivary gland inflammation through EMT. This may occur from acinar cells that are transformed to fibroblastic cells generating new fibrous tissue replacing epithelial cells, resulting in more production of immature collagens. 31 Nevertheless, the effect of EMT on changes in collagen fibers needs to be further investigated in salivary gland diseases. Moreover, studying the differences in noncollagenous proteins among salivary gland diseases would be interesting to give us better understanding in the salivary gland pathologies.

## Conclusion

This study exploits the simple advantage of picrosirius red staining combined with polarized microscope to characterize collagen fibers in submandibular salivary gland diseases. Inflammation, and benign and malignant tumors of salivary gland diversely affect the amounts and maturity of collagen. The results provide a useful explanation in different histopathologies and behaviors among various salivary gland diseases.
